# Hydrogel-derived non-precious electrocatalysts for efficient oxygen reduction

**DOI:** 10.1038/srep11739

**Published:** 2015-07-01

**Authors:** Bo You, Peiqun Yin, Junli Zhang, Daping He, Gaoli Chen, Fei Kang, Huiqiao Wang, Zhaoxiang Deng, Yadong Li

**Affiliations:** 1 CAS Key Laboratory of Soft Matter Chemistry & Collaborative Innovation Center of Suzhou Nano Science and Technology, Department of Chemistry, University of Science and Technology of China, Hefei, Anhui 230026, China; 2 Center of Advanced Nanocatalysis (CAN-USTC), University of Science and Technology of China, Hefei, Anhui 230026, China; 3 Department of Chemistry, Tsinghua University, Beijing, 100084, China

## Abstract

The development of highly active, cheap and robust oxygen reduction reaction (ORR) electrocatalysts to replace precious metal platinum is extremely urgent and challenging for renewable energy devices. Herein we report a novel, green and especially facile hydrogel strategy to construct N and B co-doped nanocarbon embedded with Co-based nanoparticles as an efficient non-precious ORR catalyst. The agarose hydrogel provides a general host matrix to achieve a homogeneous distribution of key precursory components including cobalt (II) acetate and buffer salts, which, upon freeze-drying and carbonization, produces the highly active ORR catalyst. The gel buffer containing Tris base, boric acid and ethylenediaminetetraacetic acid, commonly adopted for pH and ionic strength control, plays distinctively different roles here. These include a green precursor for N- and B-doping, a salt porogen and a Co^2+^ chelating agent, all contributing to the excellent ORR activity. This hydrogel-based process is potentially generalizable for many other catalytic materials.

The rapidly increasing global energy consumption coupled with the critical issue of climate change has triggered tremendous research efforts towards clean and renewable energy sources^1^. In response, fuel cells and metal-air batteries have been the focus of intense research due to their high energy density, high efficiency, and negligible emission of harmful gases^2^,^3^. The foremost limitation in these energy-transforming appliances is the kinetically sluggish four-electron oxygen reduction reaction (ORR) on costly, scarce and vulnerable platinum-based cathode catalysts, which prohibits their large-scale commercialization^4^,^5^,^6^. As a consequence, considerable efforts have been aimed at developing non-precious metal oxide (CoO, Co_3_O_4_, MnCo_2_O_4_ and MnO_2_), metal-nitrogen complex (Fe-N_x_, Co-N_x_ and FeCo-N_x_), and metal-free heteroatom (N, B, S, P and F) doped nanocarbons as potential substitutes for platinum and its alloys in electrocatalytic ORR^5^,^6^,^7^,^8^,^9^,^10^,^11^,^12^. Thanks to the superior conductivity, large specific surface area, and high mechanical strength of graphene, carbon nanotubes (CNTs), and nanoporous carbon, many innovative electrocatalysts have been developed^6^,^7^,^8^,^11^. However, problems plaguing such catalysts still exist, including microstructural non-homogeneity (due to heterogeneous synthetic routes) and tedious multistep preparations^13^,^14^,^15^,^16^,^17^,^18^,^19^.

To improve the structural homogeneity of an ORR catalyst, bottom-up processes have been developed for catalyst fabrications using specially synthesized organic compounds as building blocks to form the catalyst structures^9^,^20^,^21^,^22^,^23^,^24^. Although the resulting non-precious catalysts sometimes exhibit outstanding ORR performance, the expensive and tedious chemical syntheses will impede a large-scale production of the catalysts. Also, toxic precursors (such as NH_3_, aniline, pyridine, pyrrole or melamine) are usually involved in the synthesis of the molecular precursors, which decrease the environmental benignity of the catalyst fabrication process. In addition, ionic liquids (ILs) have proved to be excellent precursors to heteroatom-doped nanocarbons with promising electrocatalytic ORR activities^25^,^26^,^27^. Unfortunately, only very few cyano- or nitrile-containing aprotic ILs can be used for this purpose. The high cost associated with the IL-derived catalysts may finally hinder their commercialization^28^. Therefore, it is advantageous and highly desirable to develop a facile, cheap and green strategy towards high performance non-precious ORR electrocatalysts.

Since the first discovery by Wichterle *et al.* in the early 1950s^29^, hydrogels, a class of three dimensional hierarchically cross-linked hydrophilic polymers with superior biocompatibility, good biodegradability and tunable porous structure have been investigated for a wide range of biomedical and pharmaceutical applications, including contact lenses, tissue engineering, diagnostics and drug delivery^30^,^31^,^32^,^33^,^34^,^35^. Specially, agarose-based hydrogel is most frequently used for analytical and preparative electrophoretic separations of DNA and proteins^36^,^37^,^38^ owing to its various advantages including low cost, negligible DNA and proteins adsorption, and very easy gel casting process^30^. Other attractive properties of agarose gel include high mechanical strength even at very low concentrations (as low as 0.15%)^30^, and excellent tolerance to extreme pH and ionic strength as well as high concentrations of chemical denaturants. We consider these properties of the agarose hydrogel especially suited for the preparation of carbon-based nanocatalysts towards electrochemical ORR via a hydrogel-based process described below.

Herein, we report a homogeneous hydrogel-based bottom-up strategy to build three dimensional porous carbon-supported non-precious electrocatalysts. As illustrated in Fig. 1, this process starts with a facile gelation of an agarose hydrogel containing cobalt (II) acetate, TBE (Tris/Boric acid/EDTA; Tris = tris(hydroxymethyl) aminomethane, EDTA = ethylenediaminetetraacetic acid) buffer, and agarose, followed by a freeze-drying and a carbonization of the gel to produce N and B co-doped nanocarbon embedded with Co-based nanoparticles for electrocatalytic ORR. The TBE buffer not only controls the gel pH but also provides a coordinating interaction with Co^2+^ to suppress its hydrolysis and alleviate the sintering or agglomeration of the Co-based nanoparticles during carbonization. In the meantime, the TBE buffer acts as N and B sources to achieve ORR-active N- and B-dopings of graphitized carbon, and as salt porogen to achieve high porosity and large specific surface area of the final product. The as-obtained hydrogel-derived non-precious electrocatalyst (HNPC, hydrogel-derived non-precious catalyst) shows homogeneous microstructure, moderate heteroatom contents (12.6% Co, 5.26% N, and 3.26% B), high specific surface area (432 m^2^ g^−1^) and large mesopores (average pore diameter of 6.4 nm) with molecular accessibility, resulting in excellent four-electron ORR performance comparable to commercial Pt/C catalysts. This potentially general hydrogel-based strategy is inexpensive, environmentally benign and easily scalable, opening up a new catalytic material-oriented application of hydrogels. It is reasonable to expect that this unique strategy can be extended to other metal oxide or metal catalysts supported on N, B co-doped nanocarbons for energy-related applications.

## Results

### Catalyst synthesis

**Figure 1:**
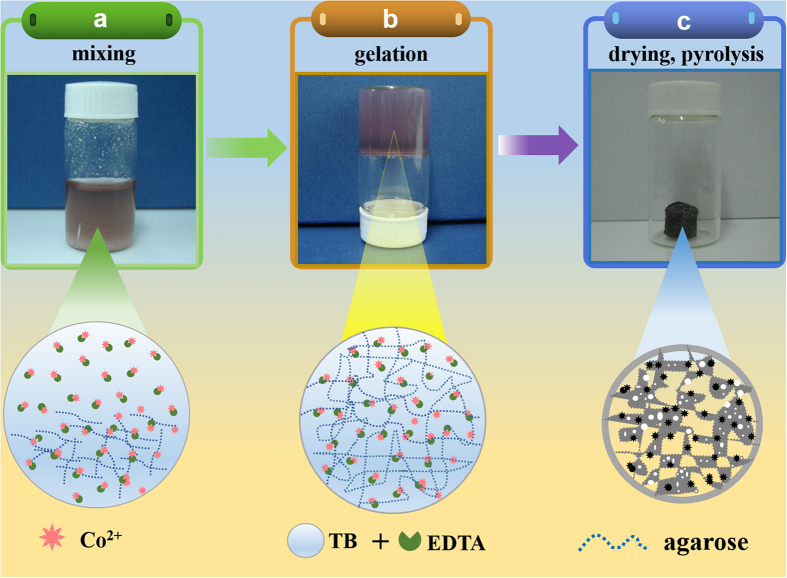
Synthesis of HNPC materials. (**a**) Three-component mixture of TBE buffer (Tris/Boric acid/EDTA), cobalt (II) acetate and agarose; (**b**) Heating up and cooling down the mixture to form a hydrogel containing homogeneously distributed functional chemical components; (**c**) Freeze-drying and thermal carbonization of the hydrogel result in ORR active HNPC materials.

**Figure 2:**
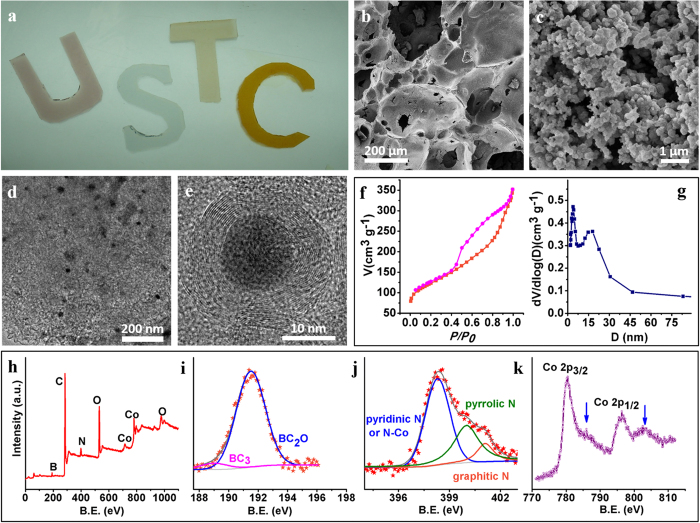
Physical characterizations of HNPC. (**a**) Photographs of the agarose hydrogels containing Co, Ni, Mn and Fe salts, corresponding to letters U, S, T and C, respectively. (**b**–**e**) SEM (**b**, **c**) and TEM (**d**, **e**) images of HNPC at different magnifications. (**f**, **g**) N_2_ sorption isotherms (**f**) and pore size distribution (**g**) of HNPC. (**h**–**k**) XPS survey spectrum (**h**), and high resolution B 1s (**i**), N 1s (**j**) and Co 2p (**k**) spectra.

Hydrogel-derived non-precious electrocatalyst (HNPC) was prepared by freeze-drying and subsequent carbonization of a TBE (Tris/Boric acid/EDTA) buffered agarose gel containing cobalt (II) acetate, as shown in Fig. 1 (see Methods for more details). After mixing the three components of TBE buffer, cobalt (II) acetate and agarose, the color of the mixture changed from pink to magenta due to the formation of a Co^2+^ -EDTA complex (Supplementary Figure S1, UV-vis data). Note that, at this step, agarose appeared as a powdery solid that did not dissolve in the TBE buffer (Fig. 1a). Upon heating to boiling, a viscous “solution” (molten state of a hydrogel) was formed, which was then rapidly gelated to form an agarose hydrogel after cooling to room temperature (Fig. 1b). The resulting gel block was freeze-dried and subjected to a thermal treatment to convert the gel into a carbonized product (HNPC) with an absolute (oven) yield of ~52% (Fig. 1c and Supplementary Figure S2, Thermogravimetric Analysis). The above process was very simple and generalizable. By using different metal salts, agarose hydrogels with easily tunable metal precursor compositions were obtained (Fig. 2a, letters U, S, T and C corresponded to Co, Ni, Mn and Fe hydrogels, respectively), which could be investigated for different functions and applications in the future.

### Physical characterizations

Powder X-ray diffraction (XRD), scanning electron microscopy (SEM), transmission electron microscopy (TEM), and X-ray photoelectron spectroscopy (XPS) were employed to probe the structural and compositional details of the as-obtained HNPC materials. The samples showed three broadened XRD peaks at 2θ = 26.2°, 43.5° and 44° (Supplementary Figure S3). The peaks at 26.2° and 43.5° corresponded to X-ray reflections from (002) and (100) planes of a graphitized carbon structure^39^. Compared with a Co-free carbon monolith prepared by a similar way (without adding cobalt (II) acetate, other components were kept the same as in HNPC), the HNPC exhibited a much stronger (002) reflection of a graphite structure, implying a Co-catalyzed graphitization during the thermo-induced carbonization^20^,^39^. Due to the relatively low cobalt content (12.6 wt% detected by ICP-AES, inductively coupled plasma atomic emission spectrometry) and very fine particle diameter (see TEM data in the following discussions), only the (111) diffraction of metallic Co (PDF 15-0806) at 2θ = 44° was resolvable as a very weak peak.

SEM images of a carbonized gel revealed an interesting interconnected carbon framework featuring hierarchical open pores (Fig. 2b,c). The low-magnification image in Fig. 2b indicated the presence of abundant macropores with a continuous size distribution in the micrometer range. A closer inspection of the macropore walls in a high-magnification SEM image revealed a mesoporous structure composed of stacked nanoparticles (Fig. 2c). In the case of a Co-free carbon monolith (Supplementary Figure S4), there was no such mesoporosity of its macropore walls, in sharp contrast to the Co-containing HNPC. Such a dramatic difference could be related to a Co-catalyzed graphitization during the carbonization process^20^. TEM images further confirmed the porosity of the as-obtained HNPC material, with the Co-based nanoparticles sparsely and uniformly embedded in the porous carbon matrix. In Fig. 2d (also see Supplementary Figure S5), Co-based nanoparticles with a uniform size ranging from 10 to 16 nm were clearly observable. High magnification TEM imaging indicated that most of the nanoparticles were tightly encapsulated by multiple layers of concentric carbon shells due to Co-catalyzed graphitization (Fig. 2e)^20^, which was in accordance with the SEM results (Fig. 2c). In contrast, the Co-free carbon monolith exhibited an amorphous structure with negligible graphitic order (Supplementary Figure S4c). The much higher degree of graphitization was expected to provide a better conductivity favorable for electrochemical applications^5^,^6^,^7^,^8^. Selected-area electron diffraction (SAED) gave multiple rings that were readily indexable as (111), (200) and (220) reflections of metallic Co (Supplementary Figure S5d), consistent with the XRD result (Supplementary Figure S3). High-resolution TEM (HRTEM) image of a randomly picked nanoparticle revealed a lattice fringe of 0.20 nm (Supplementary Figure S5f), corresponding to the (111) plane of face-centered cubic (fcc) Co (PDF: 15-0806). These data suggested the existence of metallic Co products embedded in HNPC.

Nitrogen sorption isotherm (Fig. 2f) and the calculated pore size distribution (Fig. 2g) based on the sorption data gave a specific surface area of 432 m^2^ g^−1^ and an average pore diameter of 6.4 nm, respectively. The high specific surface area, large pore size, and three-dimensional (3D) interconnected structure of the HNPC material would facilitate the mass transport of ORR-relevant species and provide a high accessibility to catalytically active sites, which is critical to promote ORR electrocatalysis^14^,^16^,^17^,^21^.

X-ray photoelectron spectroscopic (XPS) analysis revealed the presence of B, C, N, O, and Co in the HNPC materials (Fig. 2h). The B content was determined to be 3.26% by ICP-AES. The N and C contents were found to be 5.26% and 69.53%, respectively, based on a combustion elemental analysis. According to literature reports, these doped heteroatoms are potential active sites for electrochemical ORR^2^,^40^. The high-resolution B1s spectrum (Fig. 2i) was deconvoluted into two peaks assignable to BC_3_ (189.1 eV) and BC_2_O (191.5 eV)^11^. Similarly, the high-resolution N1s spectrum (Fig. 2j) could be fitted by three sub-peaks corresponding to pyridinic N (398.7 eV), pyrrolic N (400.0 eV) and graphitic (quaternary) N (401.1 eV)^10^. In addition, catalytically inert N-B configuration (397.9 eV) was not observed^40^. The peak at 398.7 eV might also include a contribution from nitrogen atoms bound to metal cobalt (N-Co), due to the small binding energy difference between N-Co and pyridinic N^41^. Co 2p_3/2_ and Co 2p_1/2_ peaks appeared at 780.5 and 796.5 eV (Fig. 2k), indicating the presence of Co_3_O_4_^42^. Concomitantly, two easily resolvable satellite peaks at 786.3 and 803.0 eV, characteristic of Co^2+^ on the octahedral site of rocksalt CoO, revealed the mixed Co valences in the Co-containing HNPC^42^. High-resolution Co 2p_3/2_ and O1s spectra confirmed the presence of metal Co, Co-N and Co-O, respectively (Supplementary Figure S6). Combining the XRD, SAED, HR-TEM and XPS evidences, the presence of metal Co, CoO and Co_3_O_4_ in HNPC, was possible which is similar to carbon-supported Co-based heterogeneous catalysts^19^.

### Electrocatalytic evaluation

**Figure 3:**
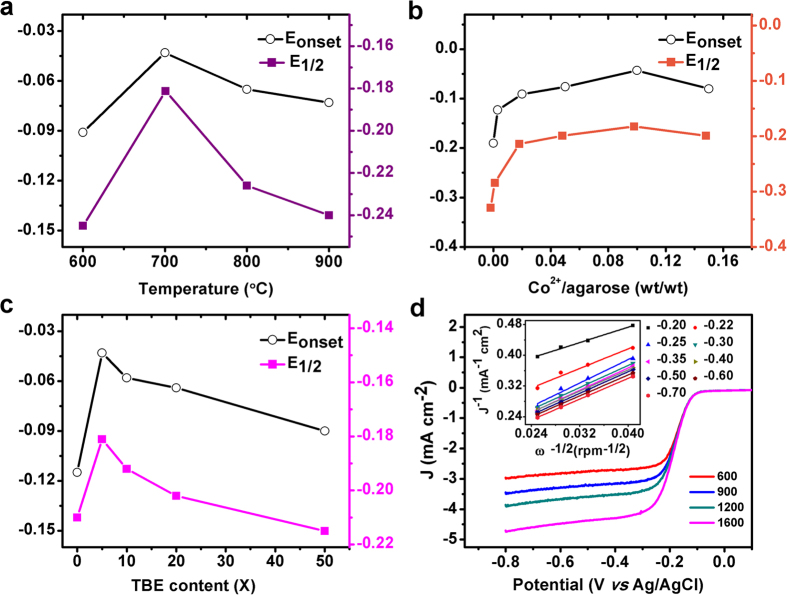
Optimization of synthetic conditions for HPNC. (**a**–**c**) Dependence of the ORR E_onset_ and E_1/2_ potentials (*vs* Ag/AgCl, based on RDE voltammetric data at 1600 rpm) on different catalyst preparation parameters, including (**a**) carbonization temperature, (**b**) cobalt ion/agarose mass ratio, and (**c**) TBE content. (**d**) ORR polarization curves (at different rotation speeds) of the HNPC composite prepared under optimized condition. Inset in (**d**) shows Koutecky-Levich plots at different potentials. For all RDE data, catalyst loading was ~200 μg cm^−2^, potential scan rate was 10 mV s^−1^ and the electrolyte was 0.1 M KOH.

Onset potential (E_onset_) and half-wave potential (E_1/2_) of a steady linear sweep voltammetric (LSV) curve on a rotating disk electrode (RDE) in an O_2_-saturated 0.1 M KOH electrolyte were employed by us to evaluate the ORR activities of the HNPC catalysts, similar to previous research^5^,^6^,^7^,^8^,^9^,^10^,^11^,^12^,^13^,^14^,^15^,^16^,^17^,^18^,^19^,^20^,^21^,^22^,^23^,^24^,^25^,^26^,^27^,^28^. To achieve the best catalytic performance, the synthetic conditions were optimized. The resulting catalyst was dispersed in a 0.2% ethanol solution of Nafion, which was then pipetted onto a polished glassy carbon electrode. Note that the recorded RDE curves were all corrected for the background currents to yield net ORR currents (See Supplementary Figure S7a). Fig. 3a–c showed the influences of carbonization temperature and the concentrations of cobalt (II) acetate and TBE buffer on the ORR activities of as-produced HNPC catalysts. It could be inferred from Fig. 3a that a carbonization temperature of 700 °C was optimal for the best ORR performance. The initial cobalt/agarose mass ratio also had a significant influence on the catalytic properties of the final product. As shown in Fig. 3b, a volcano-shaped dependence of catalytic activity on cobalt content was observed. Both E_1/2_ and E_onset_ experienced a steep positive shift after the introduction of cobalt but finally reached a maximum at 10% cobalt/agarose mass ratio. In addition, we found that 5 × TBE in the gel recipe resulted in the highest ORR activity compared to other TBE concentrations, as shown in Fig. 3c.

We therefore took the optimal parameters to prepare HNPC catalysts for further electrochemical characterizations. Cyclic voltammetric (CV) data of HNPC showed a pronounced peak at −0.175 V vs Ag/AgCl in an O_2_-saturated solution, in contrast to a N_2_-saturated solution, implying a prominent ORR activity (Supplementary Figure S8). The steady-state ORR polarization curves of the HNPC catalyst exhibited well-defined diffusion-limiting currents (−0.8 to −0.28 V, *vs* Ag/AgCl) following a mixed kinetic-diffusion region at different RDE rotating speeds (Fig. 3d). In addition, the catalysts prepared in different batches had a good reproducibility (Supplementary Figure S9). The mass activity of the HNPC at −0.15 V was 4.6 A g^−1^, higher than that of a cobalt porphyrin-derived Co-N-C catalyst (1.5 A g^−1^)^20^. Moreover, linearity of Koutecky-Levich plots and the near parallelism of the fitting lines suggested first-order reaction kinetics with respect to dissolved O_2_ and potential-independent electron transfer numbers (Fig. 3d inset)^5^,^6^. The slopes of the Koutecky-Levich plots^5^,^6^ revealed a four electron (n ≈ 3.8e in our case) pathway for the oxygen reduction process catalyzed by the HNPC.

**Figure 4:**
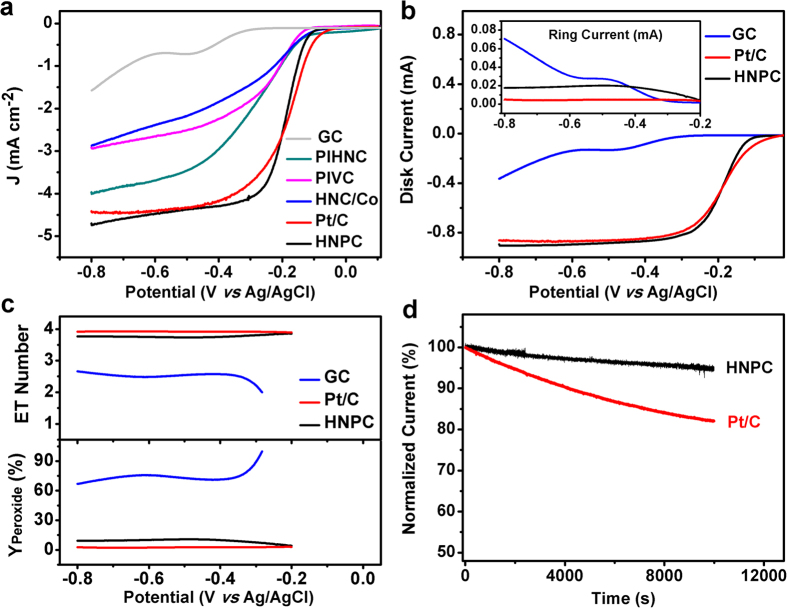
ORR activity of HNPC in comparisons with various control samples. (**a**) RDE polarization curves of bare glassy carbon electrode (GC), HNPC, Pt/C, HNC/Co, PIHNC, and PIVC (see text). The electrochemical tests were performed with a glassy carbon RDE at 1600 rpm in O_2_-saturated 0.1 M KOH with a potential scan rate of 10 mV s^−1^. (**b**) RRDE current curves of HNPC, Pt/C, and bare GC in O_2_-saturated 0.1 M KOH at 1600 rpm with a potential scan rate of 5 mV s^−1^. Inset shows corresponding ring currents. (**c**) Electron transfer (ET) numbers (n) (top) and peroxide yields (bottom) of the HNPC and Pt/C catalysts derived from the RRDE data in (**b**), as a function of disk electrode potential. (**d**) Chronoamperometric responses of the HNPC and Pt/C catalysts in O_2_-saturated 0.1 M KOH at a constant potential of −0.4 V (*vs* Ag/AgCl) and an electrode rotation speed of 1600 rpm. The loadings of the non-precious catalysts and the commercial Pt/C were ~200 μg cm^−2^ and 75 μg cm^−2^, respectively.

It is commonly accepted that elemental composition, dispersion homogeneity of active sites, specific surface area, structural porosity, and conductivity will greatly influence the activity of an ORR catalyst^2^,^4^,^5^,^6^,^7^,^8^,^9^,^10^,^11^,^12^,^13^,^14^,^15^,^16^,^17^,^18^,^19^,^20^,^21^,^22^,^23^,^24^,^25^,^26^. Therefore, we speculated that the high specific surface area, large pore size, dispersion homogeneity of catalytic sites, and good conductivity of the HNPC catalyst could account for its superior ORR activity. To provide some evidences for this assumption, control samples were prepared and their ORR activities were compared with the HNPC catalyst. Firstly, to verify the importance of dispersion homogeneity of the active sites, we prepared a sample by post-impregnating a purely hydrogel-derived nanocarbon (HNC) with cobalt (II) acetate in 5 × TBE buffer followed by a freeze-drying and a carbonization process (termed as PIHNC, post-impregnated hydrogel-derived nanocarbon). Secondly, to reveal the positive effect of the 3D porosity of HNPC on ORR, we used commercial carbon black (Vulcan XC-72) as a support to prepare another control (PIVC, post-impregnated Vulcan carbon) via a post impregnation process similar to PIHNC. Additionally, a physical mixture of an agarose gel-derived Co-free carbon material and Co-based nanoparticles (HNC/Co) obtained by a direct calcination of cobalt (II) acetate in 5 × TBE buffer under the same conditions was also prepared and evaluated for its ORR activity. Apparently, the bare glassy carbon electrode (GC) showed the most negative E_onset_ and the smallest current density, implying negligible catalytic activity (Fig. 4a). Moreover, all the three controls exhibited much lower catalytic activities than the HNPC material, as judged from the polarization curves in Fig. 4a. It was evident that the HPNC material showed more positive E_onset_ and E_1/2_ potentials and significantly higher diffusion-limiting current. To highlight the activity of the HNPC sample, a commercially available Pt/C catalyst (20% Pt on Vulcan XC-72 carbon black) was chosen as a benchmark to make a strict comparison between the two catalysts. Although E_onset_ of the HNPC was slightly lower (more negative) than Pt/C, their E_1/2_ potentials were very close to each other (−0.181 V *vs* −0.177 V for HNPC and Pt/C, respectively). Besides, the HNPC showed a steeper rise of the ORR current before reaching a diffusion limit and a slightly higher diffusion current from −0.2 V to −0.8 V, in comparison with Pt/C. These data supported the fact that the much cheaper and more easily fabricated HNPC catalyst had an ORR activity very close to the commercial Pt/C catalyst (see Supplementary Table S1 for a comparison with other nonprecious catalysts).

The specificity of the HNPC-catalyzed four-electron oxygen reduction towards the production of H_2_O was studied by a rotating ring-disk electrode (RRDE) method. An oxidization potential was applied to the Pt ring electrode so that peroxide by-products generated during the reduction of O_2_ on the catalyst-loaded disk electrode could be monitored. It was found that the ring current (due to the oxidation of peroxide by-products generated on the central disk electrode) was much smaller for HNPC than a bare glassy carbon (GC) (Fig. 4b inset). In addition, the disk current curves were very similar for both the HNPC and Pt/C catalysts (Fig. 4b). Based on the RRDE data, the peroxide yield of the HNPC-loaded electrode was calculated to be <10.8%. These data corresponded to an average electron transfer number (n) of 3.81 from −0.2 to −0.8 V (Fig. 4c), consistent with the Koutecky-Levich plots (Fig. 3d inset). The RRDE measurements indicated a mainly four electron (n ≈ 3.81) pathway for the HNPC catalyst, which was close to Pt/C (peroxide yield <2.6% and average n ≈ 3.92) (Fig. 4c).

The durability of an ORR catalyst is another practical issue that determines the life of an O_2_ electrode in a real battery. The stability of the HNPC catalyst was then tested by RDE chronoamperometry (Fig. 4d) under steady state mass transport conditions. The ORR current catalyzed by the HNPC decreased by only 5% over 10 000 s of continuous operation. In sharp contrast, the commercial Pt/C catalyst showed a 18% decrease of its ORR current during the same test. These results unambiguously evidenced a superior stability of the HNPC catalyst in an alkaline electrolyte, which could be related to its robust structure and the strong bonding between the Co-based nanoparticles and surrounding graphitic layers. Such an excellent durability of the HNPC material delivers a high performance ORR catalyst that guarantees a long-term use with negligible activity loss.

## Discussion

We attribute the advantageous electrocatalytic activity of HNPC to its unique nanoarchitecture formed during the hydrogel-based process. (1) The agarose gel offers a 3D hierarchically interconnected framework with microsize porosity in the final products, which promotes the accessibility to N, B doped sites and Co-based nanoparticles for ORR related species; (2) The EDTA component in the TBE buffer forms a stable chelate with Co^2+^, responsible for a suppressed sintering or reduced agglomeration of Co-based nanoparticles (by controlling the release of cobalt ion before carbonization and forming an isolating carbon matrix after pyrolysis) during a high-temperature carbonization reaction, due to the so-called molecular confinement effect^13^; (3) The TBE buffer salt serves as a “salt porogen” to introduce additional porosity, further boosting the specific surface area of the catalyst^26^,^27^; (4) The nontoxic Tris, EDTA, and Boric acid in the TBE buffer function as environmentally friendly N and B sources for an effective N, B co-doping into a graphitic lattice; It is noteworthy that the co-doping of N with higher electronegativity (χ = 3.04) and B with lower electronegativity (χ = 2.04) in comparisons with C (χ = 2.55) results in a unique electronic structure of the nanocarbons so that enhanced catalytic activity may be expected^40^; (5) The hydrogel-based gelation-carbonization process results in a homogeneous distribution of the catalytic “centers” (N, B, and Co-based nanoparticles) in the HPNC for ORR, which is highly beneficial for the enhanced catalytic activities.

We have carried out a series of control experiments to provide experimental supports for the above hypotheses. The molecular confinement effect of EDTA has been examined by removing EDTA from the gel recipe, and the resulting catalyst shows aggregated Co-based nanoparticles with a particle size increased to ~30 nm (Supplementary Figure S10). Also, the porogenic role of TBE salts has been clearly demonstrated by taking away TBE from the hydrogel. As shown in supplementary Figure S11, in the absence of both TBE and cobalt (II) acetate, a nanocarbon material (HNC, purely hydrogel-derived nanocarbon) with significantly reduced surface area and total pore volume (240 m^2^ g^−1^ and 0.19 cm^3^ g^−1^, respectively) has been obtained. This forms a sharp contrast to another control sample where TBE is present (HNC-TBE, surface area and total pore volume of 497 m^2^ g^−1^ and 0.24 cm^3^ g^−1^, respectively). In addition, the pure agarose gel-derived nanocarbon material (HNC, see above) and a commercially available conductive carbon support (Vulcan XC-72) have been employed to prepare the catalysts via a post-impregnation process. The resulting catalysts have been found to contain severely aggregated Co-based nanoparticles (Supplementary Figure S12) with poor ORR activity (Fig. 4a). This is easily understandable because the hydrogel-based scheme is well-suited to generate a homogeneous distribution of cobalt (II) acetate in the gel matrix, and finally lead to uniformly embedded Co-based nanoparticles in the 3D porous carbon framework.

## Conclusion

In summary, we have developed a novel, low cost, green, and especially facile hydrogel-based strategy to constitute N and B co-doped nanocarbon containing Co-based nanoparticles for efficient ORR electrocatalysis with high activity close to commercial Pt/C. The TBE buffer not only allows the formation of EDTA-Co^2+^ complex to suppress the hydrolysis of Co^2+^ and alleviate the sintering/agglomeration of Co-based nanoparticles, but also acts as green N and B precursors and a salt porogen. The agarose hydrogel provides a unique carbon source for the formation of highly conductive graphitic carbon during a thermal treatment under the catalytic action of cobalt compounds. The resulting hydrogel-derived non-precious catalyst shows homogeneous microstructure, moderate heteroatom contents (12.6% Co, 5.26% N, and 3.26% B), high specific surface areas (432 m^2^ g^−1^) and large mesopores (average pore diameter of 6.4 nm), leading to excellent four-electron ORR activity. The extreme simplicity and low cost of the hydrogel-based method endows it with a high promise for industrial scale catalyst manufacturing. Moreover, such a process should also be generalizable to produce other types of carbon-based nanocatalysts for various energy-related applications, which will be pursued in our following up research.

## Methods

### Synthesis of HNPC with high catalytic activity

First, 0.4 g agarose powder was added into 20 mL 5 × TBE buffer (445 mM Tris base, 445 mM boric acid, and 10 mM EDTA) containing 0.169 g of cobalt (II) acetate. Upon heating to boiling, the agarose was dissolved to form a viscous and clear solution. This solution was allowed to cool down to room temperature so that a homogeneous hydrogel containing all the necessary chemical components was obtained. The agarose-based hydrogel was then freeze-dried, and the dehydrated monolith was subjected to a thermal treatment at 700 °C for 2 h in an argon flow. The as-prepared carbon materials were washed by deionized water and dried at 80 °C in vacuum. In our control experiments, the hydrogel compositions (cobalt (II) acetate, TBE buffer and agarose) as well as the carbonization temperature were individually altered to show their different contributions to the final HNPC product.

### Characterizations

Transmission electron microscopy (TEM) images were taken on a JEM 2100 F microscope (JEOL, Japan) operated at 200 kV. Scanning electron microscopy (SEM) imaging was carried out on a Sirion 200 microscope (FEI, USA) operated at 5 kV. Nitrogen sorption isotherms were obtained at 77 K with a Micromertics ASAP 3020 analyzer (Micromertics, USA). Before each measurement, the sample was degassed in vacuum at 200 °C for at least 5 h. The Brunauer-Emmett-Teller (BET) method was used to calculate the specific surface areas of the samples. The Barrett-Joyner-Halenda (BJH) model was utilized to analyze pore size distributions, based on which total pore volumes could be obtained. Powder X-ray diffraction (XRD) data were collected with a MiniFlex 600 diffractometer (Rigadu, Japan) using Cu Kα radiation (40 kV, 15 mA). The X-ray photoelectron spectra (XPS) were recorded on an ESCALab MKII X-ray photo-electron spectrometer using Mg Kα radiation as an exciting source. The Co and B contents were determined with an Optima 7300 DV ICP-AES (PerkinElmer, USA). The N and C contents were analyzed with a VarioELIII element analyzer (Elementar Analysensysteme GmbH, Germany).

### Catalyst evaluation

The catalyst ink was prepared by ultrasonically mixing 4 mg of pre-grounded catalyst powder in 1 mL of 0.2% Nafion (Sigma-Aldrich) ethanol solution for 30 min to form a homogeneous suspension of the catalyst particles. 10 μL of the catalyst ink was pipetted onto a polished glassy carbon rotational disk electrode (RDE), corresponding to a catalyst loading of ~200 μg cm^−2^. For comparison purpose, commercially obtained Pt/C catalyst (20 wt%, Johnson Matthey) was loaded to reach an electrode coverage of ~75 μg cm^−2^. Electrochemical measurements with RDE (rotating disk electrode) and RRDE (rotating ring-disk electrode) were carried out with a three-electrode cell system. A computer-controlled electrochemical workstation (CHI760E, Chenhua Inc., China) was employed for all electrochemical tests. A glassy carbon RDE (PINE, 5 mm diameter) and a platinum ring glassy carbon disk RRDE (PINE, 5.6 mm diameter) loaded with different electrocatalysts were used as the working electrodes, an Ag/AgCl electrode (3 M KCl) as the reference electrode, and a Pt wire as the auxiliary electrode. Oxygen reduction reaction (ORR) activities of different catalysts were characterized in an O_2_ saturated (pure O_2_) 0.1 M KOH electrolyte at room temperature. The potential was scanned from +0.2 to −0.8 V at a scan rate of 10 mV s^−1^ and various rotation speeds from 600 to 1600 rpm. While for the RRDE tests, the scan rate and rotation speed were 5 mV s^−1^ and 1600 rpm, respectively. The onset potential was obtained from the first derivative of a polarization curve (where a derivative current deviated from the baseline by 3 times of baseline noise). Each sample was tested 3 times to avoid any incidental errors.

## Additional Information

**How to cite this article**: You, B. *et al.* Hydrogel-derived non-precious electrocatalysts for efficient oxygen reduction. *Sci. Rep.* **5**, 11739; doi: 10.1038/srep11739 (2015).

## Supplementary Material

Supplementary Information
