# Supplementary material for: Hydrogel-derived non-precious electrocatalysts for efficient oxygen reduction
Source: Sci Rep. 2015 Jul 1;5:11739. doi: 10.1038/srep11739 (PMC4486930; doi:10.1038/srep11739)
Supplement: Supplementary Information [file srep11739-s1.pdf]

Hydrogel-derived non-precious electrocatalysts for efficient oxygen reduction

Bo You,[1] Peiqun Yin,[2] Junli Zhang,[1] Daping He,[2] Gaoli Chen,[1] Fei Kang,[1] Huiqiao Wang,[1] Zhaoxiang Deng[1,2*] and Yadong Li[2,3]

[1] CAS Key Laboratory of Soft Matter Chemistry & Collaborative Innovation Center of Suzhou Nano Science and Technology, Department of Chemistry, University of Science and Technology of China, Hefei, Anhui 230026, China
[2] Center of Advanced Nanocatalysis (CAN-USTC), University of Science and Technology of China, Hefei, Anhui 230026, China
[3] Department of Chemistry, Tsinghua University, Beijing, 100084, China

Correspondence and requests for materials should be addressed to Z.D.
(*email: zhxdeng@ustc.edu.cn)

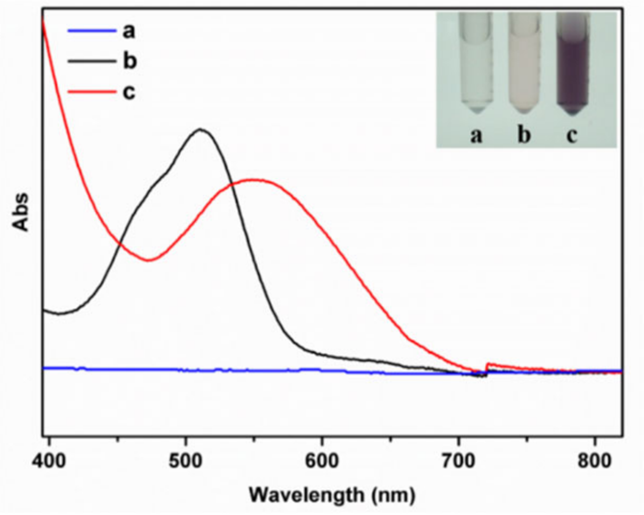

Fig. S1. Visible absorbance spectra of (a) 5× TBE buffer, (b) Cobalt acetate aqueous solution, and (c) Cobalt acetate in 5× TBE buffer solution.

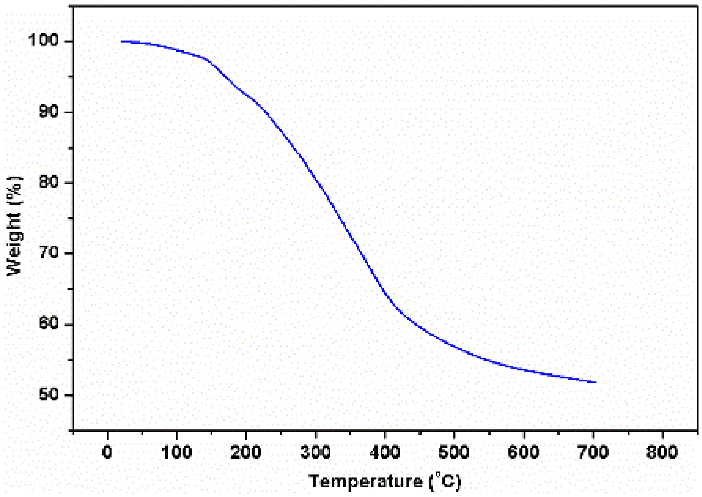

Fig. S2. Thermogravimetric analysis (TGA) of a freeze-dried Co-containing hydrogel in argon.

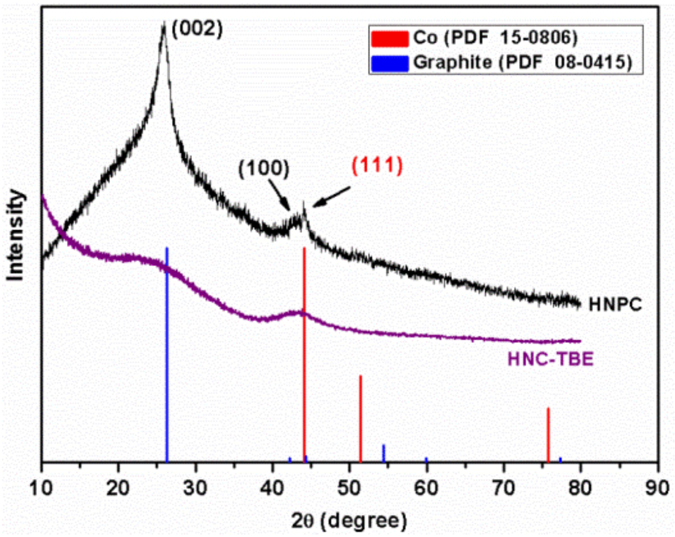

Fig. S3. Powder XRD patterns of a hydrogel-derived non-precious catalyst (HNPC) in the presence of cobalt acetate and 5×TBE, and purely hydrogel derived nanocarbon in the presence of 5×TBE (HNC-TBE).

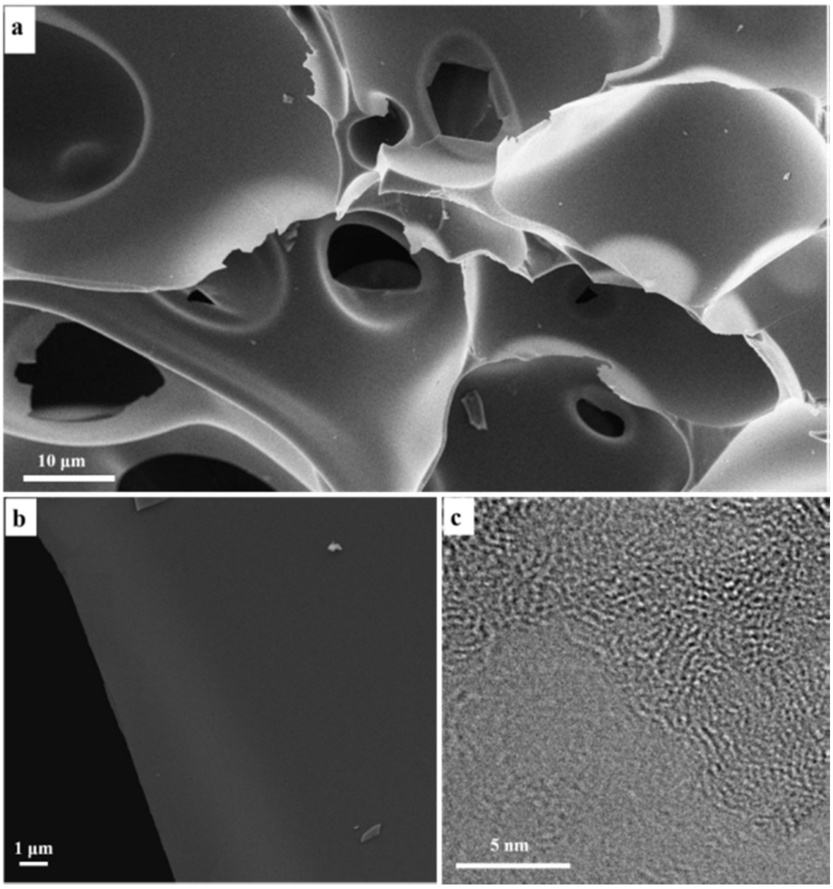

Fig. S4. (a, b) SEM and (c) HR-TEM images of cobalt-free carbon monolith (HNC-TBE) prepared in the absence of cobalt acetate, while other components including agarose and TBE remained the same as HNPC.

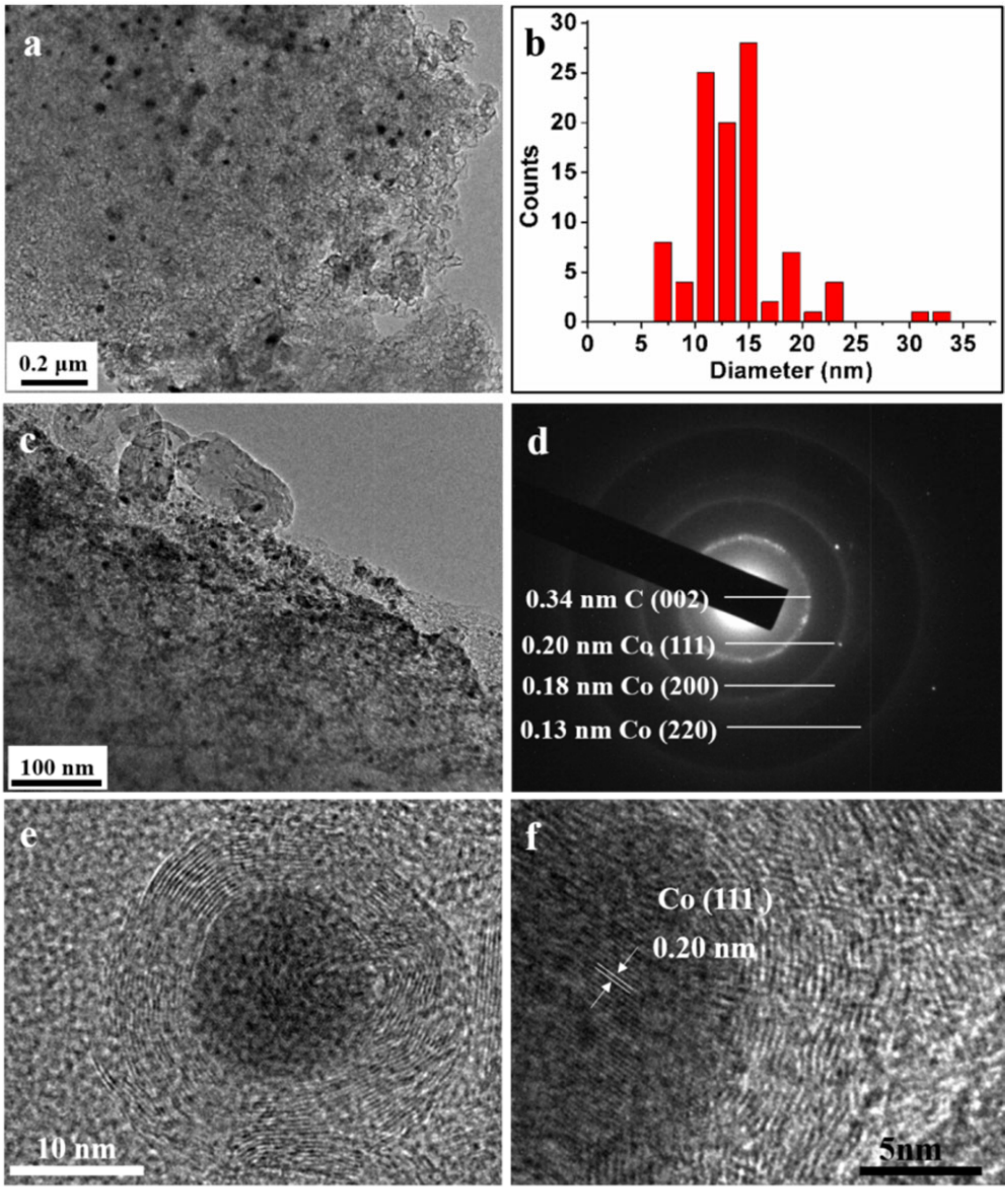

Fig. S5. (a, c e and f) TEM images of low and high magnifications; (b) Statistical size distribution of the Co-based nanoparticles (in a); (d) Electron diffraction (ED) rings of an HNPC sample as shown in (c), which was readily indexed to the (111), (200) and (220) reflections of Co (PDF: 15-0806) and the (002) reflection of C.

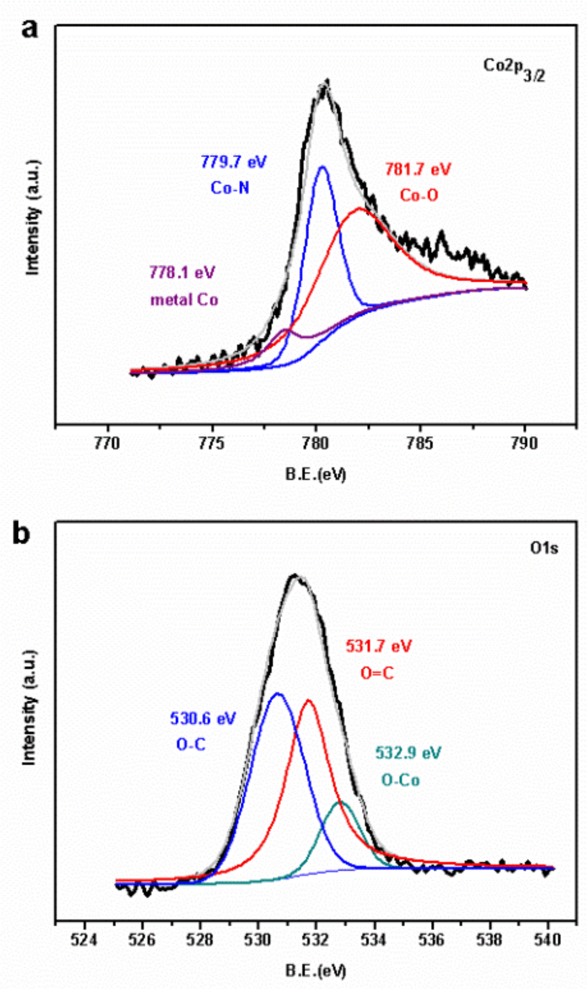

Fig. S6. High-resolution XPS spectra of (a) Co $2p_{3/2}$ and (b) O1s of an HNPC.

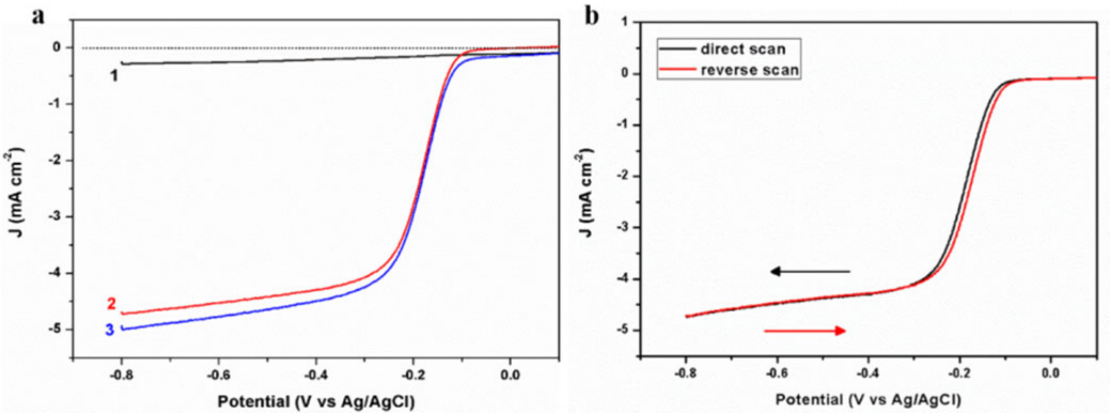

Figure S7. (a) RDE polarization curves of HNPC at 1600 rpm in O_2-free (curve 1) and O_2-saturated (curve 3) 0.1 M KOH. The curve 2 in (a) was obtained by subtracting curve 3 from curve 1. (b) RDE voltammetric curves of HNPC corresponding to cathodic and anodic potential scans in an O_2-saturated solution, which showed a negligible difference. Potential scan rates were 10 mV/s in all cases.

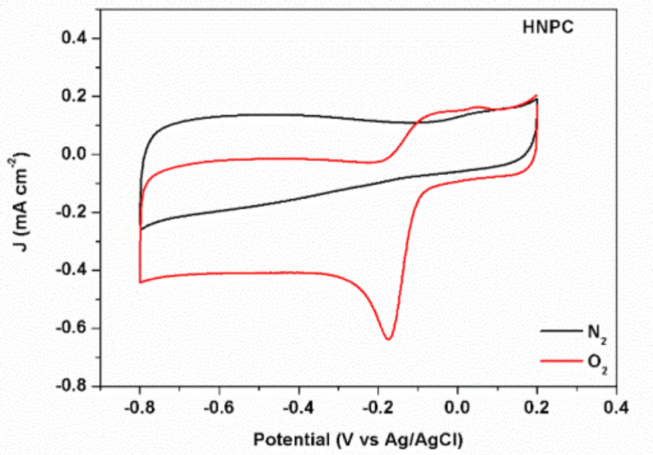

Figure S8. CV curves of HNPC in O_2-free (black curve) and O_2-saturated (red curve) 0.1 M KOH. The scan rate was 10 mV/s.

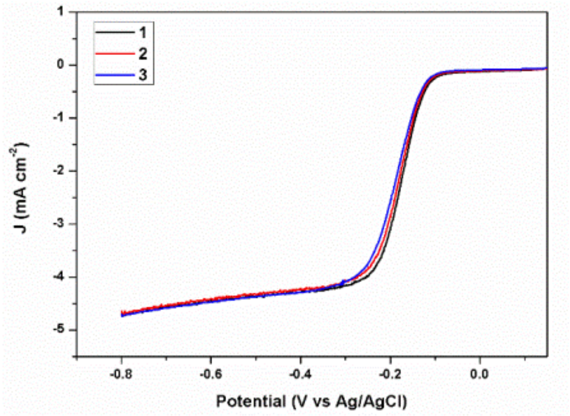

Figure S9. RDE polarization curves of HNPC prepared in different batches in an O_2-saturated 0.1 M KOH at 1600 rpm. The half-wave potentials were -0.177, -0.174, and -0.172 V, respectively, with a relative derivation of less than 2%.

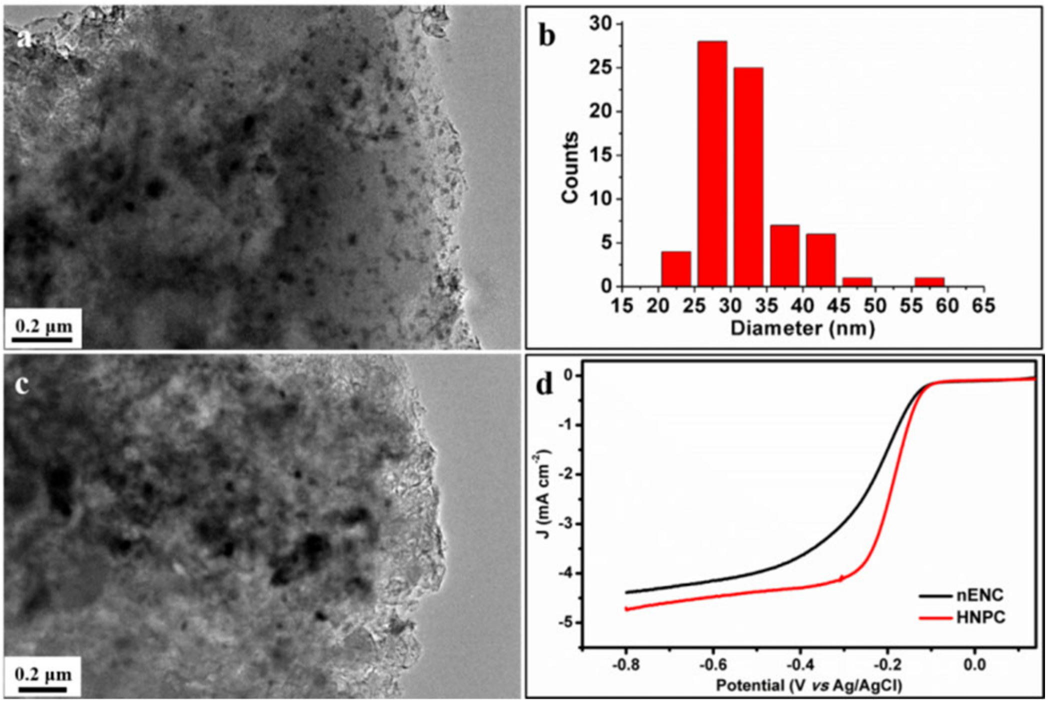

Figure S10. (a, c) TEM images of a control HNPC sample prepared in the absence of EDTA (named as nENC); (b) size distribution of Co-based nanoparticles in (a); (d) RDE polarization curves of HNPC in comparison with the control sample (nENC, EDTA was not added) in an O_2-saturated 0.1 M KOH solution. Electrode rotation speed was 1600 rpm, and potential scan rate was 10 mV s^{-1}.

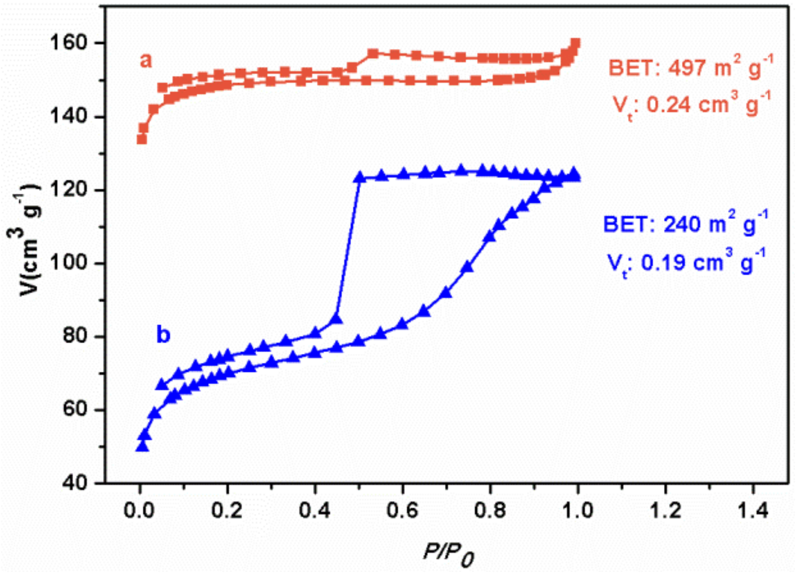

Figure S11. N_2 sorption isotherms of (a) HNC-TBE (prepared from a Co-free agarose hydrogel in 5×TBE buffer), and (b) HNC (hydrogel-derived nanocarbon material, both TBE and cobalt acetate were omitted from the gel recipe). The specific surface area and total pore volume of HNC-TBE were 497 $m^2\ g^{-1}$ and 0.24 $cm^3\ g^{-1}$, respectively, which were significantly larger than the HNC material (specific surface area and total pore volume of 240 $m^2\ g^{-1}$ and 0.19 $cm^3\ g^{-1}$, respectively).

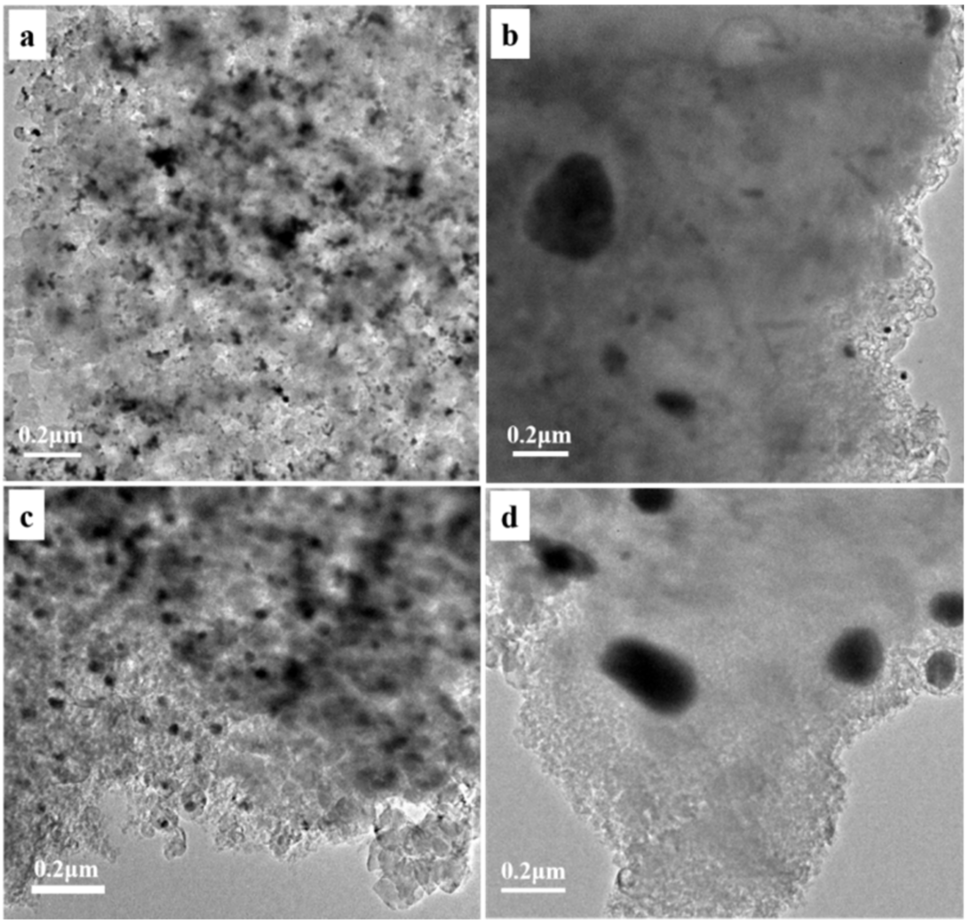

Figure S12. (a, b) TEM images of PIHNC (post-impregnated HNC) and (c, d) PIVC (post-impregnated Vulcan XC-72 carbon) samples.

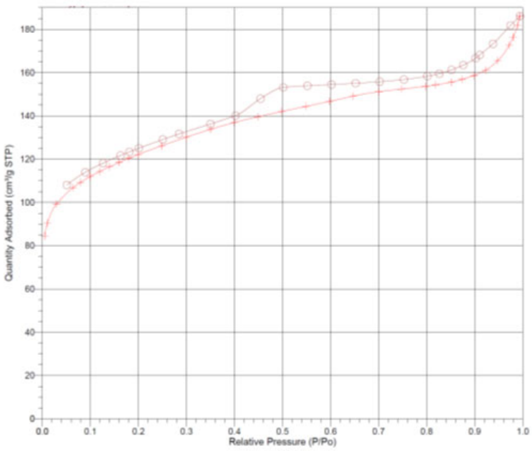

Carbonization: 600ºC; BET: 424 m^2/g

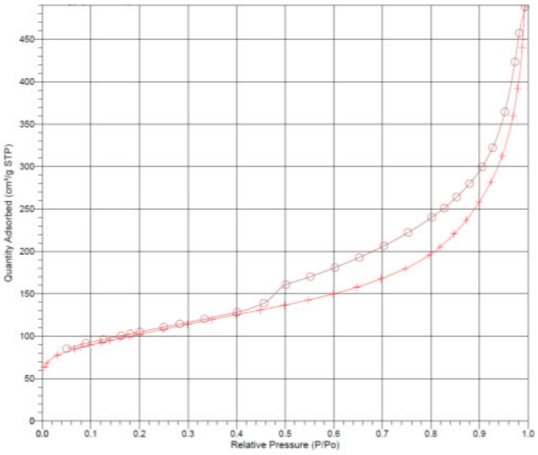

Carbonization: 800ºC; BET: 362 m^2/g

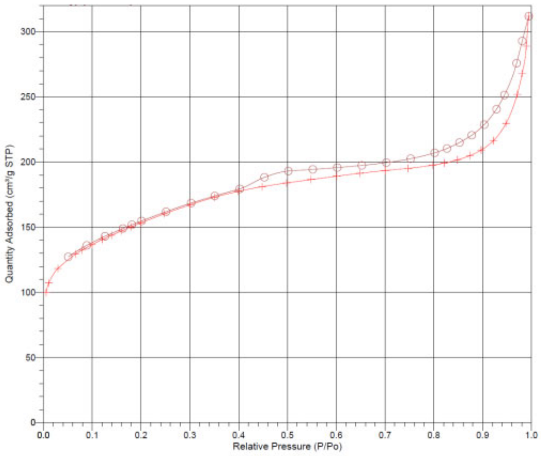

Co/agarose mass ratio: 0.15; BET: 539 m^2/g

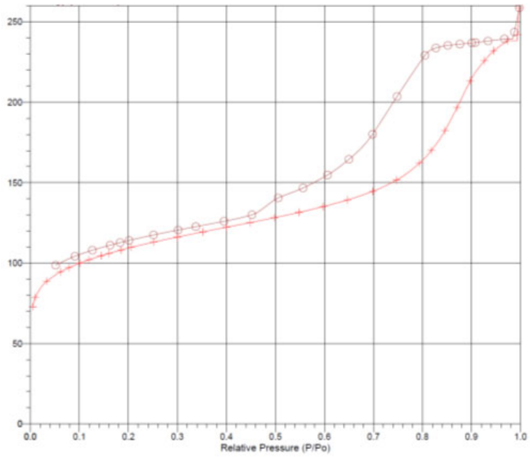

TBE: 20×; BET: 379 m^2/g

Figure S13. Extra N_2 sorption data corresponding to some typical samples in Figure 3. According to the data in Figures S13, 2f, and S11, the respective BET surface areas were 424, 432, and 362 m^2/g for 600, 700 (optimal) and 800 ºC treatments; and 497, 432 and 539 m^2/g for 0%, 10% (optimal) and 15% Co/agarose mass ratios. Either carbonization temperature or Co content did not seem to significantly affect the surface areas. The halfwave and onset potentials in Figures 3a,b could therefore reflect the different inherent activities of these catalysts for comparison purpose. The TBE content did affect the surface areas of the samples. BET surface areas were 240, 432 and 379 m^2/g for 0x, 5x (optimal) and 20x TBE, respectively. This is understandable since TBE could behave as a salt porogen. The different surface areas of the catalysts prepared with different TBE concentrations might account partially for their different activities (especially for the sample without TBE). Note that to achieve a high surface area of a catalyst is always an important pursuit in order to maximize catalyst utilizations.

Table S1. A comparison of ORR activities among different non-precious metal-based electrocatalysts including HNPC in 0.1 M KOH.

**Electrocatalysts**	**$E_{1/2}$ (V) (vs Ag/AgCl)**	**Specific mass activity (A/g) at -0.15 V**	**Reference**
HNPC	-0.18	4.6	*This work*
$NiCo_2O_4$-rGO	-0.32	0.3	*Adv. Mater.* **2014**, 26 2408.
GNC-Co	-0.17	4.0	*Angew. Chem. Int. Ed.* **2013**, 52, 12105.
N-OMMC-G	-0.22	1.3	*Adv. Mater.* **2013**, 25, 6226.
Co-N-C	-0.18	1.5	*Adv. Mater.* **2014**, 26, 1450.
